# Pencil‐Drawn Generator Built‐in Actuator for Integrated Self‐Powered/Visual Dual‐Mode Sensing Functions and Rewritable Display

**DOI:** 10.1002/advs.202206467

**Published:** 2023-01-10

**Authors:** Wansong Gu, Peidi Zhou, Wei Zhang, Zhiling Luo, Luzhuo Chen

**Affiliations:** ^1^ Fujian Provincial Key Laboratory of Quantum Manipulation and New Energy Materials College of Physics and Energy Fujian Normal University Fuzhou 350117 P. R. China; ^2^ Fujian Provincial Collaborative Innovation Center for Advanced High‐Field Superconducting Materials and Engineering Fuzhou 350117 P. R. China; ^3^ Fujian Provincial Engineering Technology Research Center of Solar Energy Conversion and Energy Storage Fuzhou 350117 P. R. China; ^4^ Institute of Smart Marine and Engineering Fujian University of Technology Fuzhou 350108 P. R. China

**Keywords:** actuator, multi‐functional, photo‐thermoelectric, self‐powered, visual sensing

## Abstract

Multifunctionality is important to the development of next‐generation actuators and intelligent robots. However, current multi‐functional actuating systems are achieved based on the integration of diverse functional units with complex design, especially lacking in multi‐mode sensing and displaying functions. Herein, a light‐driven actuator integrated with self‐powered/visual dual‐mode sensing functions and rewritable display function is proposed. The actuator demonstrates a bending curvature of 0.93 cm^−1^ under near‐infrared light irradiation. Meanwhile, by embedding a pencil‐drawn graphite generator and thermochromic materials, the actuator also provides two independent sensing functions. First, owing to the photo‐thermoelectric effect of graphite, the actuator spontaneously outputs a self‐powered voltage (Seebeck coefficient: 23 µV K^−1^), which can reflect the deformation trend of actuator. Second, color changes occur on the actuator during deformation, which provide a visual sensing due to the thermochromic property. Furthermore, the actuator can be utilized as a rewritable display, owing to the integrated color‐memorizing component. Intelligent robots, switches, and smart homes are further demonstrated as applications. All of them can spontaneously provide self‐powered and visual sensing signals to demonstrate the working states of actuating systems, accompanied by rewritable displays on the actuators. This study will open a new direction for self‐powered devices, multi‐functional actuators, and intelligent robots.

## Graphical abstract

A multi‐functional light‐driven actuator is proposed, which is integrated with dual‐mode sensing functions and rewritable display function. The actuator is fabricated by embedding a pencil‐drawn graphite generator and a screen‐printing thermochromic layer. The dual‐mode sensing functions include the self‐powered sensing function and the visual sensing function. Furthermore, the actuator can be utilized as a rewritable display.

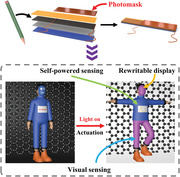

## 1 Introduction

Nowadays, the application of flexible actuators in wearable devices,^[^ ^1^, ^2^ ^]^ intelligent robots,^[^ ^3^, ^4^ ^]^ and bionics^[^ ^5^, ^6^ ^]^ has attracted much attention due to their excellent actuation property and compatibility. With the deepening of actuator research, traditional single‐function actuators can no longer meet existing needs. One of the current research trends in the development of actuators is multi‐functionalization, especially integrating sensing functions.^[^ ^7^, ^8^, ^9^ ^]^ Nevertheless, the requirements for sensing functions are becoming more and more demanding with the technological development. As far as the current research is concerned, there are still some problems with multi‐functional actuators. For example, an external power source is always required for sensing,^[^ ^10^ ^]^ which needs a computer to convert electrical signals into identifiable information and the preparation process is complicated.

The developments of triboelectric nanogenerator (TENG) and piezoelectric nanogenerator (PENG) have led to many self‐powered devices. Touching nanogenerator with external pressure can convert mechanical energy into electrical energy. Through external circuit transmission, the force applied to nanogenerator can be monitored in real‐time, so as to monitor the activities of the operator.^[^ ^11^, ^12^, ^13^ ^]^ As self‐powered sensing devices, TENGs and PENGs have been widely used in pressure sensing,^[^ ^14^ ^]^ environmental monitoring,^[^ ^15^ ^]^ health monitoring,^[^ ^16^ ^]^ and other fields. However, TENGs and PENGs have a disadvantage that they need dynamic touch to generate power. A thermoelectric generator (TEG) can solve this problem. It has the capability to generate electricity without direct contact. The principle of electricity generation in TEG is due to the temperature difference, charge carriers (electrons or holes) within a material migrate, which in turn forms an electrical potential difference.^[^ ^17^, ^18^, ^19^ ^]^ In daily life, many places have temperature difference, so TEGs have unlimited utilization prospects. In particular, thermal‐driven actuators are prepared based on the mechanism of thermal expansion, so it is advantageous to integrate the self‐powered sensing of TEG into thermal‐driven actuators.^[^ ^20^ ^]^

However, the above studies only focus on the optimization of actuators for self‐powered sensing abilities, but lack research on another crucial function: visual sensing. Many intelligent devices are given color‐switching property to achieve visual recognition of external stimuli,^[^ ^21^, ^22^ ^]^ which will facilitate operator manipulation and reduce the integration of electronic sensing systems. Actuator, as one of the intelligent devices, is also integrated with color‐switching property by many researchers. Therefore, the operator can distinguish the different working states of the actuator by color‐switching. For example, some researchers reported hydrogel actuators with deformation and reversible color conversion under stimulation.^[^ ^23^, ^24^ ^]^ Inspired by chameleon, structural‐color actuators were presented, which demonstrated programmable shape transformations together with vivid color alterations.^[^ ^25^ ^]^ In addition, a thermochromic interlayer was integrated to bimorph actuators for real‐time visual signal feedback.^[^ ^26^ ^]^ However, the single color‐switching property in these studies is too monotonous, and it is difficult to display richer information on the actuator. Above all, even though some advances have been made, the exploration to achieve high‐efficiency multi‐mode sensing in one actuator is still challenging.

Here, we report a light‐driven actuator integrated with self‐powered/visual dual‐mode sensing functions, and rewritable display function. The actuator is fabricated based on graphite (G), thermochromic dye (TD), paper (P), and polyimide (PI). The graphite layer serves as a photo‐thermoelectric (PTE) generator in the actuator, which is fabricated by a pencil‐on‐paper method. When irradiated by near‐infrared (NIR) light, the actuator shows a bending actuation due to the mismatch of thermal expansion between paper and PI.^[^ ^27^, ^28^, ^29^ ^]^ At the same time, a temperature difference between the two ends of actuator is produced. Due to the PTE property of graphite, the actuator is able to generate a self‐powered electrical voltage spontaneously. Meanwhile, due to the color‐memorizing property of TD layer, the working states of the actuator can be observed by human eyes. Furthermore, complex images can be (re)written on the TD layer for information display. Based on these favorable properties of the actuator, we further demonstrated a series of intelligent systems, all of which can spontaneously output self‐powered electrical voltages and achieve visual sensing, demonstrating the working states of the actuator. This kind of actuator, which combines multi‐functions, is expected to be widely used in the fields of wearable devices, intelligent robots, smart homes, and bionic devices.

## 2 Results and Discussion

### 2.1 Fabrication and Characterization of the Actuator

**Figure 1:**
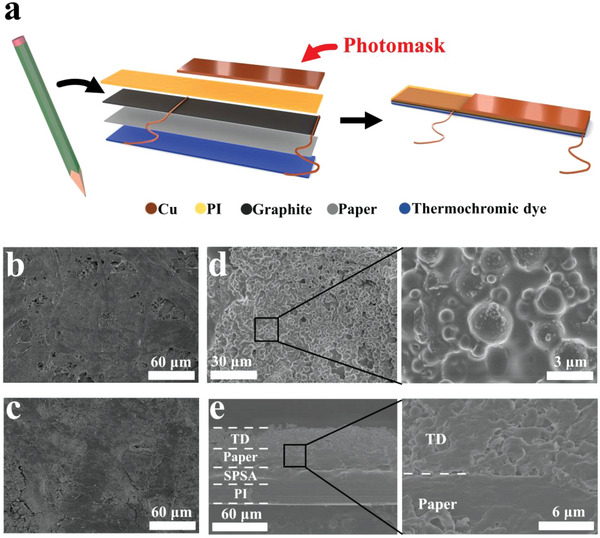
Characterization of the TDPG/PI actuator. a) Schematic diagram of the structure of TDPG/PI actuator. b) SEM image of the surface of plain paper. c) SEM image of the surface of graphite layer. d) SEM image of the surface of TD layer. e) Cross‐sectional SEM image of the TDPG/PI film.

The structure of the designed actuator is schematically illustrated in **Figure** **1**a. First, a graphite layer was coated on one side of the paper through a simple pencil‐on‐paper method, which serves as the thermoelectric (TE) layer and the photothermal layer.^[^ ^20^, ^29^ ^]^ Also, a mixed TD was coated on the other side of the paper through a screen‐printing method. Then, a flexible paper‐based TD‐P‐G (TDPG) film was first obtained. Second, two copper electrodes were embedded in the graphite layer. Third, a PI film coated with silicon pressure sensitive adhesive (SPSA) was attached to the graphite layer. Finally, the TDPG/PI actuator with a multilayer structure was obtained by attaching a piece of copper foil to cover one end of the actuator as a photomask (Figure 1a). More experimental details are described in the Experimental Section.

Figure 1b is a scanning electron microscopy (SEM) image showing the surface of plain paper, which has a porous structure. After the pencil‐on‐paper process, the pore structure is covered by graphite (Figure 1c). The surface of TD layer consists of spherical particles which are capsule balls of TD (Figure 1d).^[^ ^30^ ^]^ The cross‐sectional SEM images of the actuator are shown in Figure 1e. The film has a distinct multilayer structure and the layers are tightly bonded. Optical photos of the TDPG film and TDPG/PI actuator are shown in Figure S1a,b (Supporting Information).

### 2.2 Thermoelectric Property of the TDPG Film

**Figure 2:**
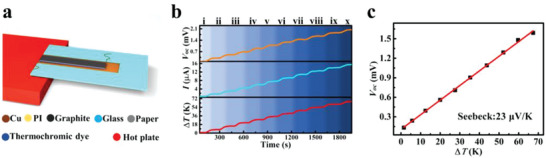
TE property of the TDPG film. a) Schematic diagram of the TE test. b) Variation of *V*_OC_, *I*_SC_, and Δ*T* of the TDPG film when heated up. c) *V*_OC_ of the TDPG film as a function of Δ*T*.

The TDPG film has TE property due to the introducing of graphite.^[^ ^31^ ^]^ The TE test diagram is presented in **Figure** **2**a. One end of the TDPG film (≈1 cm) was placed on a hot plate as a hot end, and the other end was suspended as a cold end. Meanwhile, in order to prevent the TDPG film from bending during the heating process, it was fixed on a glass frame with additional PI tapes. The PI film can withstand high temperature and has good adhesion.^[^ ^32^ ^]^ The structure of the TDPG film in the TE test is shown in Figure S2 (Supporting Information). Two copper electrodes were fixed at two ends of the TDPG film. When the hot plate was powered on, the temperature of the hot end of TDPG film increased and produced a temperature difference to the cold end. Owing to the TE effect, a self‐powered voltage would be generated between the hot and cold ends of the TDPG film. In order to measure the Seebeck coefficient, ten stages of temperature were set by the hot plate, and each stage lasts 180 s. Additionally, infrared thermal images of the TDPG film during the heating process are shown in Figure S3 (Supporting Information). The variation of the open‐circuit voltage (*V*_OC_) and short‐circuit current (*I*_SC_) of the TDPG film with the temperature difference (Δ*T*) are shown in Figure 2b. The *V*_OC_ and *I*_SC_ increase with the Δ*T* increasing, and the variation trends of *V*_OC_, *I*_SC_, and Δ*T* are consistent, which means that the *V*_OC_ and *I*_SC_ have favorable linear relations with Δ*T*. The connection between *V*_OC_ and Δ*T* is presented in Figure 2c. The calculation of Seebeck coefficient is displayed in Note S1 (Supporting Information).^[^ ^33^ ^]^ When the Δ*T* reaches 67.4 K, the *V*_OC_ is 1.59 mV. The Seebeck coefficient of the TDPG film is 23 µV K^−1^. In a word, the TDPG film is able to be a self‐powered sensor because of its TE property.

### 2.3 Color‐Switching Property of the TD‐P Film

The color‐switching property is attributed to the thermochromism of TD. In order to measure the absorbance of the TD alone, a TD layer was coated on paper by screen‐printing method to form a TD‐P (TDP) film without coating the graphite layer.

The TD is a mixture of two kinds of thermochromic dyes (ratio of 3:7), which are named as thermochromic dye A (TD‐A) and thermochromic dye B (TD‐B). The TD‐A is a color‐memorizing thermochromic dye that is composed of a color former (crystal violet lactone (CVL)), a color developer (Phenolic compound), and a solvent (aliphatic esters or aliphatic carboxylic acids). The color of TD‐A is blue at room temperature. When the TD‐A is heated over 65 °C, the CVL and the developer are dissolved and separated in the solvent. Moreover, the interaction between the CVL (electron donor) and the developer (electron acceptor) is blocked by the solvent molecule, so that the color of TD‐A changes from blue to white. Later, the CVL and the developer are dissolved in the solvent, and the solidification point of the mixture decreases. Thus, the TD‐A can keep the white color when the temperature returns to room temperature. When the TD‐A is cooled down to −20 °C, the CVL and the developer are recrystallized into the solid‐state and the interaction between them is recovered, so that the color of it returns to blue.^[^ ^34^, ^35^ ^]^

The TD‐B is a reversible thermochromic dye based on solvent, color developer, and color former compositions, which only has color‐switching property and does not have the color‐memorizing property. The use of solvent in this system is to maintain a suitable medium for interaction between color former and color developer. The solvent is in solid‐state and below the color‐switching temperature, and the interaction between color former and color developer is at minimum, which results in a colorless system. When melted, the solvent solubilizes and the interaction between the color former and the color developer is at maximum, which results in a colorful system.^[^ ^36^, ^37^, ^38^ ^]^ The melting temperature of thermochromic dye is within the sensing temperature of graphite in general. The color of TD‐B is white at room temperature, and changes to pink at 40 °C. When the TD‐B is cooled down at room temperature, the color of it returns to white.

**Figure 3:**
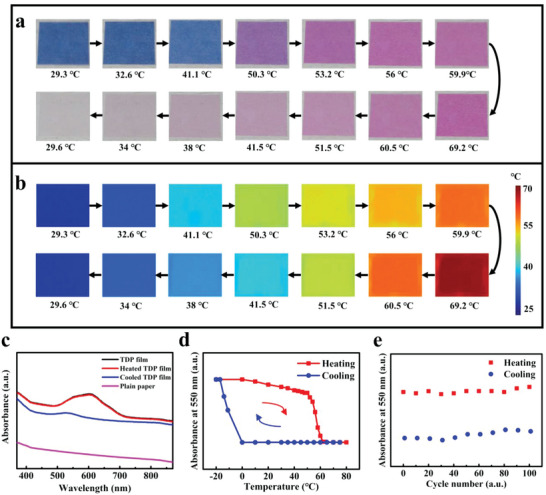
Color‐switching property of the TDP film. a) Optical photos of the TDP film heated by a hot plate under different temperature. b) Infrared thermal images of the TDP film corresponding to Figure 3a. c) Absorbance spectra of the TDP film (black line), heated TDP film (red line), cooled TDP film (blue line), and plain paper (pink line). d) Absorbance spectrum showing the hysteresis characteristic of the TDP film (at the wavelength of 550 nm). e) Absorbance spectrum of the TDP film during 100 heating/cooling cycles (at the wavelength of 550 nm).

Based on the color‐memorizing property of TD‐A and the color‐switching property of TD‐B, the color change process of TDP film is as follows. Its color is blue at room temperature. When the TDP film is heated over 50 °C, its color gradually changes from blue to pink. Then, its color changes from pink to white gradually when the temperature returns back to room temperature. The color changes are shown in **Figure** **3**a, while the corresponding infrared thermal images are shown in Figure 3b. Furthermore, when the TDP film is cooled down to −20 °C, its color returns to blue in 10 min. In a word, the color‐memorizing of TD mixture comes from the special characteristics of TD‐A.

As shown in Figure 3c, the TDP film (black line) had a strong absorption peak at the wavelength of 610 nm, so it showed blue color. When it was heated over 50 °C and back to room temperature, the strong absorption peak disappeared and its color changed from blue to pink, and then to white (blue line). When it was cooled down to −20 °C, the blue color appeared again together with the absorption peak at the wavelength of 610 nm (red line). The absorbance spectrum of the plain paper is also shown in Figure 3c (pink line) for comparison.

To further study the color‐switching property, the TDP film was heated from room temperature (20 °C) to 80 °C and then cooled down to −20 °C. Finally, it returned to room temperature (20 °C). The sample was placed at each temperature for 10 min. Then, it was placed in an ambient environment at room temperature for 5 min. After that, the absorption of TDP film was measured. The absorption intensities (at the wavelength of 550 nm) at each temperature are also shown in Figure 3d. The temperature change progress is indicated by arrows in Figure 3d. When the temperature was lower than 50 °C, the color of TDP film could almost keep white during the temperature decreasing process, exhibiting the color‐memorizing property. Only after the temperature was lower than −20 °C, the color of TDP film returned to blue. Then, its color kept blue during the temperature increasing process until the temperature reached 50 °C. When the temperature was higher than 50 °C, the color of TDP film turned to pink again.

In order to explore the repeatability of the TD, the absorption (at the wavelength of 550 nm) of the TDP film was measured over 100 cycles of heating and cooling processes. The absorbance spectrum showed no obvious decline, as shown in Figure 3e. Therefore, the TDP film has favorable repeatability in color‐switching.

It should be noted that the special color‐memorizing property of TD‐A enables the TDP film to be used as a rewritable medium for display. A TDP film (50 mm × 50 mm) was prepared to demonstrate this function, as shown in Figure S4a (Supporting Information). First, a word “pencil” was handwritten on the TDP film by using an electrothermal pen, of which the temperature was set to 80 °C (Figure S5a, Supporting Information). The word could be maintained on the TDP film, even if the pen tip left the paper surface. Then, the film was put on a hot plate with temperature higher than 50 °C, so that the color of whole TDP film turned from blue to pink, and then to white (Erase). Afterwards, the TDP film was placed in a freezer with temperature lower than −20 °C, so that the color of whole TDP film turned from white to blue (Initializing). According to Figure 3e, the TDP film has favorable repeatability and reversibility, so the operators can rewrite information on the film by using the electrothermal pen conveniently.

Furthermore, a two‐dimensional code and other complex images with high resolution can be printed on the TDP film by using a thermal printer (Figure S4b, Supporting Information). After being initialized under low temperature (< −20 °C), the TDP film can be printed repeatedly as well. In addition, the information can be retained on the TDP film for at least 360 days, showing excellent stability as an information display (Figure S5b, Supporting Information).

### 2.4 Actuation and Dual‐Mode Sensing of TDPG/PI Actuator

**Figure 4:**
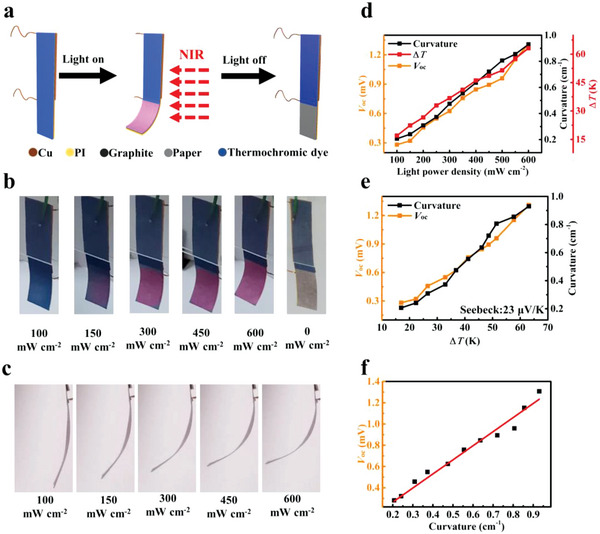
Actuation and dual‐mode sensing of the TDPG/PI actuator. a) Schematic diagram of the NIR light‐driven actuation. b) Optical photos of the actuator under different light powers (front view). c) Optical photos of the actuator under different light powers (side view). d) Δ*T*, *V*_OC_, and bending curvature of the actuator as a function of light power density. e) *V*_OC_ and bending curvature of the actuator as a function of Δ*T*. f) *V*_OC_ of the actuator as a function of bending curvature.

The TDPG/PI film with a multilayer structure is a light‐driven actuator. The test diagram is shown in **Figure** **4**a. The structure of TDPG/PI film is shown in Figure S6 (Supporting Information). As described in the Experimental Section, the upper part of actuator is fixed in a glass frame, while the lower part of it can bend freely. Therefore, when the whole actuator is irradiated by NIR light, the upper part of actuator is not affected because of the integrated photomask on the actuator. The temperature of the lower part increases due to the photothermal effect, while the temperature of the upper part remains almost unchanged. Therefore, by integrating the photomask into the actuator, there is no need for using external photomask during light irradiation, which facilitates the use of actuator.

The PI is a polymeric material with a large coefficient of thermal expansion (CTE), so it will expand under temperature rise.^[^ ^27^, ^28^, ^29^ ^]^ Meanwhile, the paper will release water molecules when the temperature increases, so the actuator will bend to the paper side during the NIR light irradiation process.^[^ ^39^ ^]^

Figure 4b shows the optical photos of the actuator under different light powers (100 to 600 mW cm^−2^). In the beginning, the whole actuator showed blue color. When the light was turned on, the temperature of the lower part of actuator increased and showed a bending actuation. When the temperature reached 50 °C or even higher, the color of the lower part would change from blue to pink. Meanwhile, the temperature of the upper part changed hardly, so there was no deformation and color‐switching in the upper part of actuator. After the light was turned off, the temperature returned to room temperature. The color of the lower part of actuator gradually turned to white and the bending deformation was restored. The color of the upper part of actuator was still blue and had no deformation. Therefore, the upper part of actuator can be used as a rewritable information display. The color‐switching of the actuator can be seen clearly in Figure 4b, and the temperature changes of the actuator can be seen from the infrared thermal images in Figure S7 (Supporting Information). Figure 4c shows the optical photos of the actuator under different light powers, in which the bending curvature change of the actuator can be seen clearly.

Especially, the TDPG/PI actuator has a dual‐mode sensing function including self‐powered sensing (electrical signal sensing) and visual sensing (color‐switching). During the light irradiation process, the temperature difference can be produced between the upper part and lower part of actuator, which leads to a self‐powered output voltage. Combining photothermal effect and TE property, the TDPG/PI actuator has the PTE property. The Δ*T*, curvature, and *V*_OC_ of the actuator were measured simultaneously under different light powers from 100 to 600 mW cm^−2^, and the results are shown in Figure 4d. The calculation of bending curvature is shown in Figure S8 and Note S2 (Supporting Information). As shown in Figure S9a (Supporting Information), when the light power density reached 600 mW cm^−2^, the Δ*T* increased 63.13 K. The corresponding *V*_OC_ reached 1.308 mV, and the bending curvature was up to 0.93 cm^−1^. Figure 4e shows that the *V*_OC_ and bending curvature of the actuator increase with the increase of Δ*T*. Moreover, the relation between *V*_OC_ and bending curvature is shown in Figure 4f. With the curvature increasing 0.1 cm^−1^, the *V*_OC_ nearly increases 0.13 mV. The Seebeck coefficient of the device measured during the actuation was 23 µV K^−1^, which was the same as the result in the previous TE test. These results show that the bending deformation of the actuator has almost no influence on the PTE property. During the deformation, the self‐powered sensing can be achieved by *V*_OC_ changing, and the visual sensing can be realized by color change. Furthermore, the rewritable information display in the actuator will be demonstrated in the following application section.

Besides, the repeatability of bending curvature and *V*_OC_ of the actuator are shown in Figure S9b (Supporting Information). One thousand actuation cycles were performed (light power of 250 mW cm^−2^). It can be seen that there is no significant decrease in bending curvature and *V*_OC_ during the repeatability test.

### 2.5 Applications of the TDPG/PI Actuators

The above results illustrate that the actuator has the self‐powered sensing function owing to the PTE property. It also has the visual sensing function and can be used as rewritable display due to the color‐memorizing property. Based on the features, three intelligent systems are developed.

**Figure 5:**
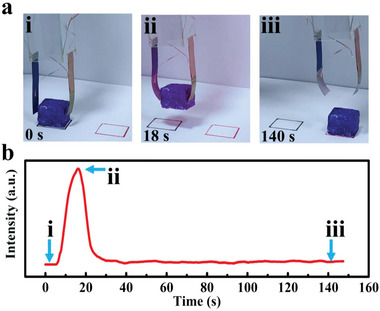
Intelligent claw based on TDPG/PI actuators. a) Optical photos showing the working process of the intelligent claw. b) Output self‐powered voltage of the intelligent claw during the operating process.

First, an intelligent claw was fabricated as a demonstration of intelligent robots. The structure of one actuator is shown in Figure S10a (Supporting Information) and the working state of claw is schematically shown in Figure S10b (Supporting Information). The grasping and releasing process of the intelligent claw is shown in **Figure** **5**a. During the NIR light irradiation, the actuators in the claw approached to an object (foam, 47 mg). The color of the lower parts of actuators changed from blue to pink, and the *V*_OC_ increased. After touching the object, the claw could pick up and move the object. When the NIR light was turned off, the actuators released the object and gradually restored. Meanwhile, the color of the lower parts of actuators changed from pink to white, and the *V*_OC_ decreased. The process of the intelligent claw grabbing and moving the object is shown in Movie S1 (Supporting Information). The *V*_OC_ changing during this process is shown in Figure 5b. The whole grabbing and moving process was fed back in real time through self‐powered voltage signal. This result shows that the actuator has dual‐mode sensing functions through self‐power voltage signal and visible color change, which overcome the shortcomings of traditional actuator.

**Figure 6:**
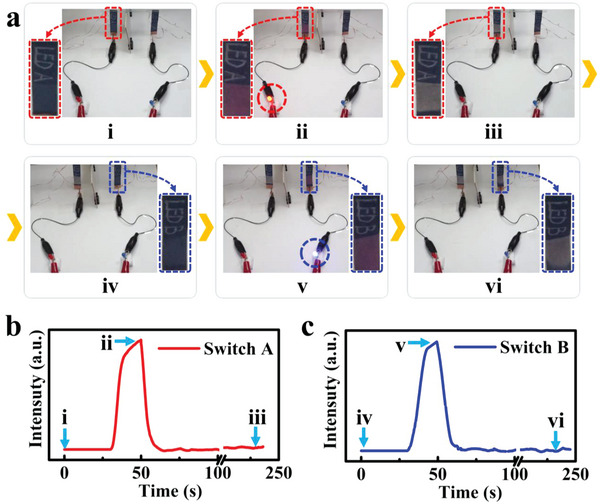
Intelligent switches based on TDPG/PI actuators. a) Optical photos showing the working process of the intelligent switches. b) Output self‐powered voltage of “Switch A” during the operating process. c) Output self‐powered voltage of “Switch B” during the operating process.

Second, an intelligent switch system was fabricated. The structure of switch is shown in Figure S11a (Supporting Information). A red LED light was controlled by “Switch A” and a blue LED light was controlled by “Switch B”. Circuit diagram of the switch system is shown in Figure S11b (Supporting Information). Besides, the working state of the switch is schematically shown in Figure S11c (Supporting Information). As shown in **Figure** **6**a, the words “LED A” was handwritten on the TD layer of the upper part of actuator. Because the photomask on the actuator would block the irradiated light, this part of actuator can be used as the rewritable information display (Figure 6a(i)). During the NIR light irradiation (light power of 250 mW cm^−2^), the lower part of actuator bent and the color changed from blue to pink. Meanwhile, the circuit was connected by the bending of actuator, and the red LED turned on (Figure 6a(ii)). Besides, as shown in Figure 6b (red line), owing to the PTE property, the *V*_OC_ increased with the bending of actuator. At last, when the NIR light was turned off, the color of the lower part of actuator changed from pink to white, and the actuator restored to the initial state. The red LED was turned off by the circuit disconnection and the *V*_OC_ decreased (Figure 6a(iii)). The process of using “LED B” was almost the same as that of using “LED A” (blue LED in Figure 6a(iv–vi)). And the corresponding self‐powered voltage signal is shown in Figure 6c. It is worth noted that the processes in Figure 6a(i–vi) and Figure 6b,c(i–vi) happened synchronously. Moreover, according to the visual sensing function of the actuator, whether the switch has been used can be deduced visually by color change. Thus, color‐switching is an important property for the actuator. Compared to the deformation of actuator, the color‐switching will be a more intuitive and perceptive signal for the direct monitoring of actuation.

**Figure 7:**
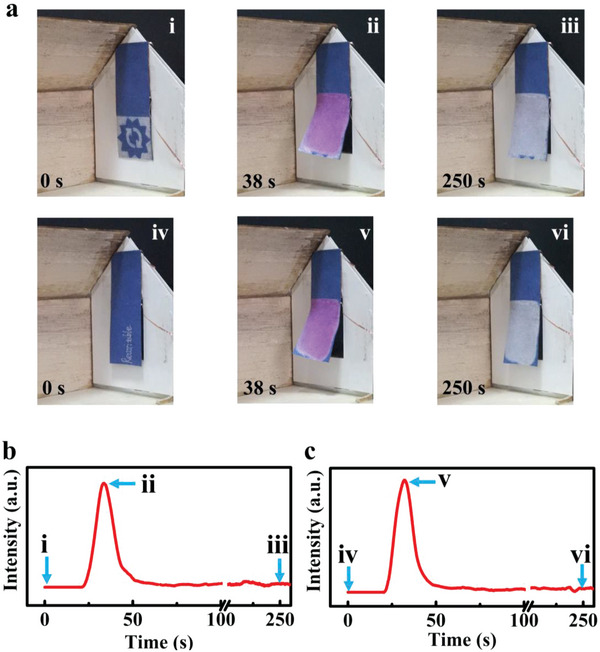
Intelligent curtain based on TDPG/PI actuator. a) Optical photos showing the working process of the intelligent curtain. b) Output self‐powered voltage of “Patterned” curtain during the operating process. c) Output self‐powered voltage of “Rewritable” curtain during the operating process.

Finally, an intelligent curtain is designed as an application demo for smart homes. The structure of the curtain is shown in Figure S12a (Supporting Information) and the working state of the curtain is schematically shown in Figure S12b (Supporting Information). As shown in **Figure** **7**a, first, an elaborate pattern was printed on the lower part of actuator by a thermal printer (Figure 7a(i)). During the NIR light irradiation, the curtain opened because of the bending actuation. The color of lower part of the curtain changed to pink and the image disappeared (Figure 7a(ii)), accompanied by the increase of *V*_OC_ (Figure 7b(ii)). When the NIR light was turned off, the intelligent curtain gradually closed. Meanwhile, the *V*_OC_ decreased (Figure 7b(iii)) and the color of lower part of the curtain changed from pink to white (Figure 7a(iii)), demonstrating self‐powered sensing and visual sensing at the same time. The opening and closing process of the “Patterned” intelligent curtain is shown in Movie S2 (Supporting Information). To show the rewritable function, the intelligent curtain was initialized. Then, a word “Rewritable” was handwritten on the initialized curtain. Similarly, another working process of the intelligent curtain under NIR light irradiation is shown in Figure 7a (iv–vi), and the corresponding output voltage signal is shown in Figure 7c (iv–vi). This application demonstrates that the actuators can not only be used in intelligent robots and electronics, but also can be used for smart homes in daily life, making the actuator close to daily life and have a wider range of applications.

## 3 Conclusion

In summary, we report a pencil‐drawn generator built‐in actuator with dual‐mode sensing functions and rewritable display function. The dual‐mode sensing functions include self‐powered sensing and visual sensing. Graphite and TD were coated on two sides of the paper respectively, and the PI film was pasted on the graphite side. The actuator demonstrates the PTE property with the Seebeck coefficient of 23 µV K^−1^. Therefore, it can generate an electrical signal without external power supply. Moreover, due to the color‐switching property of TD, the working state of actuator can be demonstrated by visual sensing without external power supply as well. Also, complex images can be (re)written on the TD layer of actuator for information display, owing to the color‐memorizing property of TD. Based on these favorable properties of actuator, we made intelligent robots, switches, and smart homes as applications. All these systems can spontaneously provide dual‐mode sensing signals to demonstrate the working states of actuating systems, accompanied by rewritable displays on the actuators.

In short, the multi‐functional light‐driven actuator not only overcomes the shortcoming of traditional actuators that cannot be self‐powered sensing, but also exhibits the visual sensing and rewritable display function. This kind of actuator that integrates multi‐functions into one device will have broad application potential in intelligent robots, wearable systems, and daily life.

## 4 Experimental Section

### Materials

The paper was a commercial product with a thickness of 44 µm. The TD‐A (blue: AKR67K01) was purchased from Shanghai M&G Stationery Inc. The TD‐B was purchased from Shenzhen East Color Technology Co., Ltd. The commercial pencil (10 B) and the PI film coated with SPSA (thickness is 40 µm) were purchased in the market.

### Fabrication of TDPG Film

The commercial paper was initially placed in a chamber at relative humidity of 70% for 5 min. Then, the humid paper was transferred on a hot plate with temperature of 100 °C for 1 min. A commercial pencil (10 B) was used to deposit graphite onto one side of the paper through the pencil‐on‐paper method. The pencil traces were drawn on the paper 500 times manually. The TD layer is made up of TD‐A and TD‐B (ratio of 3:7). By the screen‐printing method, the TD layer was printed on the other side of paper. After being dried at room temperature for 12 h, a homogeneous TD layer was obtained.

### Fabrication of TDPG Film in TE Test

Two copper‐foil electrodes were fixed at the two ends of the TDPG film. Then, the TDPG film was fixed on a glass frame with PI tape. The specific dimension of TDPG film is shown in Figure S2 (Supporting Information).

### Fabrication of TDPG/PI Actuator

The length of the whole actuator was 50 mm. The actuator includes the upper part (30 mm) and the lower part (20 mm). Two copper‐foil electrodes were embedded on the two ends of the upper part (graphite layer), in order to form an electrically conductive path. A commercial PI coated with SPSA was attached to the graphite layer (50 mm × 10 mm × 40 µm (length × width × thickness)). Then, a piece of copper foil (30 mm × 10 mm × 20 µm (length × width × thickness)) was pasted on the PI film to cover the upper part of the actuator as the photomask. The specific dimension of TDPG/PI actuator is shown in Figure S6 (Supporting Information).

### Fabrication of the Intelligent Systems for Applications

The specific dimensions of the systems are shown in Figures S10–S12 (Supporting Information).

### Characterization and Measurement

SEM images were captured by a field emission SEM (Hitachi SU8010). In the hysteresis characteristic test, the sample was placed on a hot plate to obtain the temperature higher than room temperature (20 °C). It was placed in a freezer to obtain the temperature lower than room temperature (20 °C). The sample was placed at each temperature for 10 min. Then, it was placed in an ambient environment at room temperature for 5 min. After that, the absorbance spectra of the TD layer were measured by a UV/vis/NIR spectrometer (PerkinElmer LAMBDA 950). All optical photos were captured by a digital camera (SONY ILCE 6000). A NIR light source (Philips BR125) was used for light irradiation. The light power density was measured by an infrared power meter (Linshang LS122A). The temperature was recorded by a laser sight infrared thermometer (Optris LS) with a temperature resolution of 0.1 °C. The temperature data were obtained from the TD surface. An infrared thermal imager (Fluke Ti10) was used to characterize the thermal distribution of the samples.

## Conflict of Interest

The authors declare no conflict of interest.

## Supporting information

Supporting Information

Supplemental Movie 1

Supplemental Movie 2

## Data Availability

The data that support the findings of this study are available from the corresponding author upon reasonable request.
