# Supplementary material for: Pencil‐Drawn Generator Built‐in Actuator for Integrated Self‐Powered/Visual Dual‐Mode Sensing Functions and Rewritable Display
Source: Adv Sci (Weinh). 2023 Jan 10;10(7):2206467. doi: 10.1002/advs.202206467 (PMC9982543; doi:10.1002/advs.202206467)
Supplement: Supplementary file 1 — Supporting Information [file ADVS-10-2206467-s002.pdf]

ADVANCED SCIENCE
Open Access

Supporting Information

for *Adv. Sci.*, DOI 10.1002/advs.202206467

Pencil-Drawn Generator Built-in Actuator for Integrated Self-Powered/Visual Dual-Mode Sensing Functions and Rewritable Display

*Wansong Gu, Peidi Zhou, Wei Zhang, Zhiling Luo and Luzhuo Chen**

Supporting Information

Pencil-Drawn Generator Built-in Actuator for Integrated Self-Powered/Visual Dual-Mode Sensing Functions and Rewritable Display

*Wansong Gu, Peidi Zhou, Wei Zhang, Zhiling Luo, and Luzhuo Chen**

W. Gu, Prof. W. Zhang, Dr. Z. Luo, Prof. L. Chen
Fujian Provincial Key Laboratory of Quantum Manipulation and New Energy Materials, College of Physics and Energy, Fujian Normal University, Fuzhou 350117, China
E-mail: ChenLZ@fjnu.edu.cn
W. Gu, Prof. W. Zhang, Dr. Z. Luo, Prof. L. Chen
Fujian Provincial Collaborative Innovation Center for Advanced High-Field Superconducting Materials and Engineering, Fuzhou 350117, China
W. Gu, Prof. W. Zhang, Dr. Z. Luo, Prof. L. Chen
Fujian Provincial Engineering Technology Research Center of Solar Energy Conversion and Energy Storage, Fuzhou 350117, China
Dr. P. Zhou
Institute of Smart Marine and Engineering, Fujian University of Technology, Fuzhou 350108, China

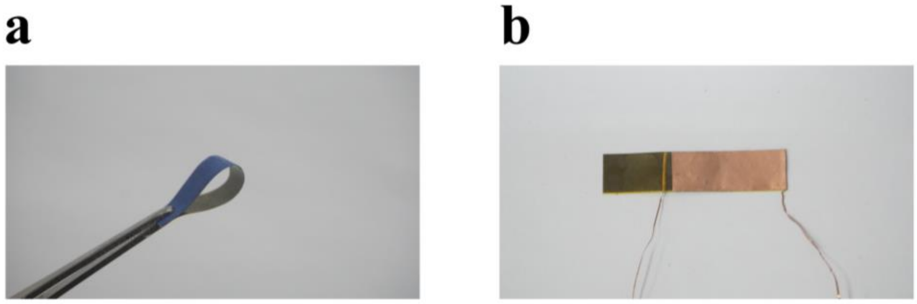

Figure S1. (a) Optical photo of the TDPG film. (b) Optical photo of the TDPG/PI actuator.

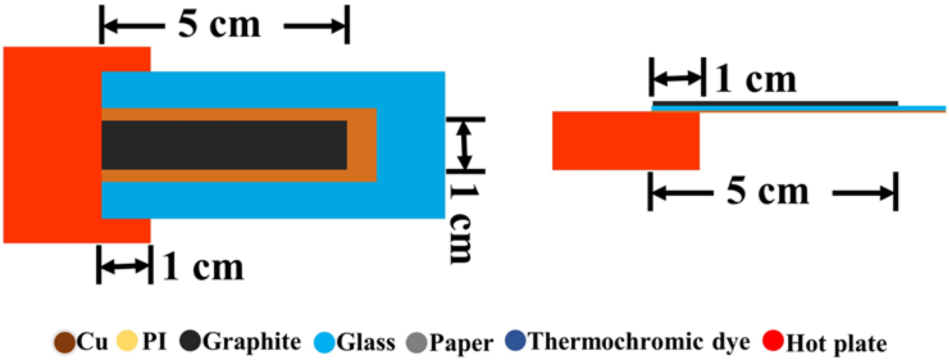

Figure S2. Structure diagram of the TDPG film in TE test.

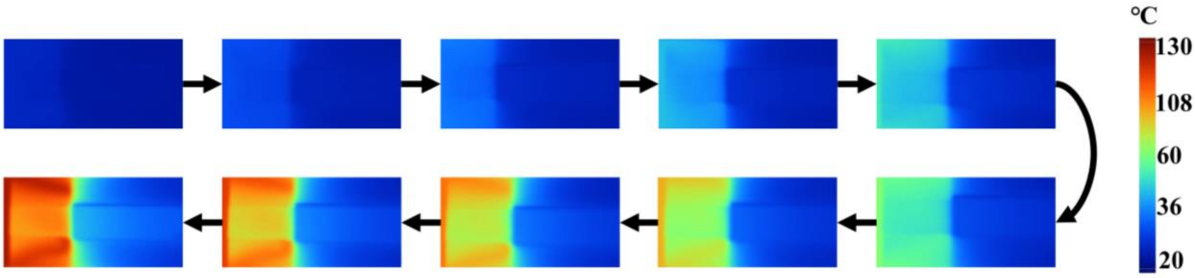

Figure S3. Infrared thermal images of the TDPG film under different temperature.

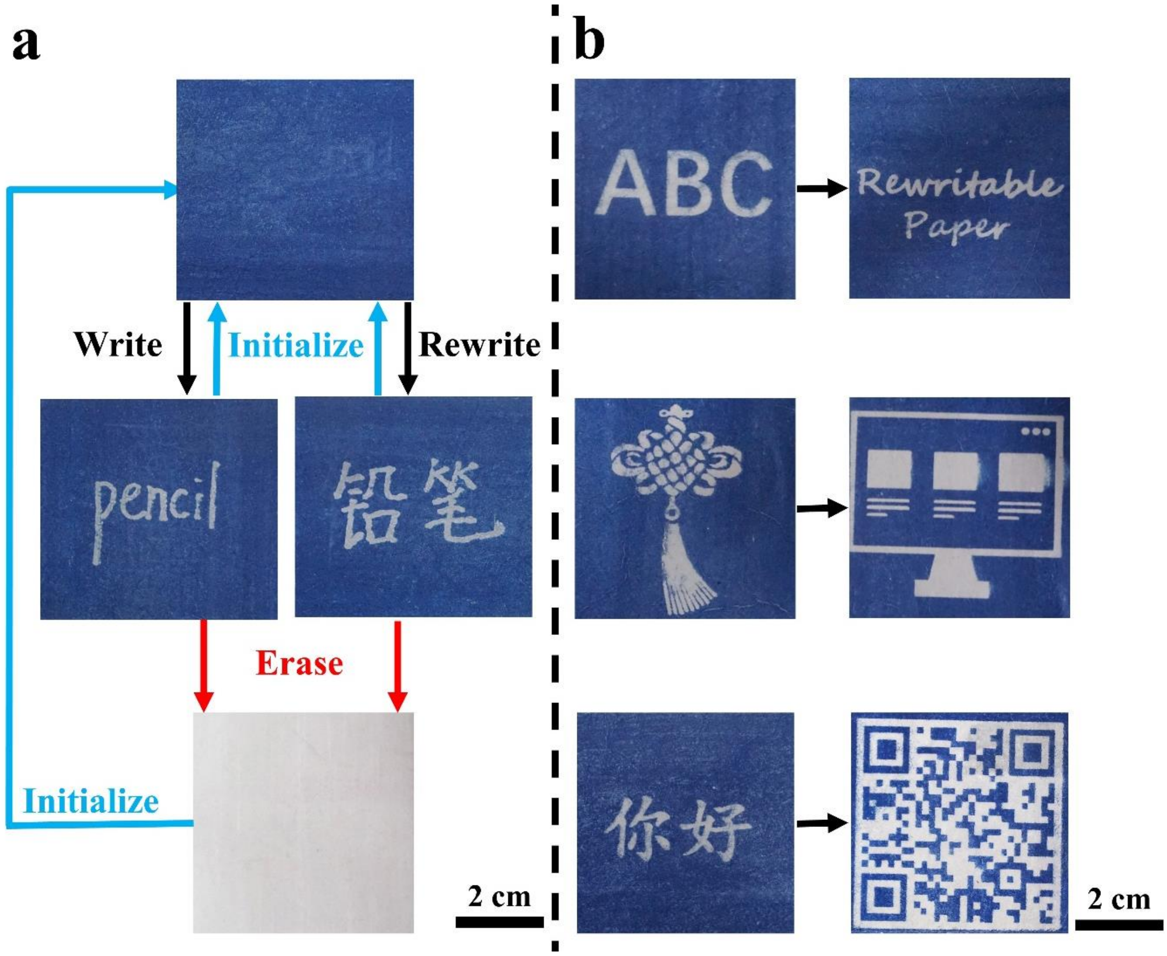

Figure S4. Rewritable function of the TDP film. (a) Optical photos showing the writing, initializing, erasing, and rewriting process of the TDP film. (b) Optical photos showing the complex patterns printed on the TDP film repeatedly by a thermal printer.

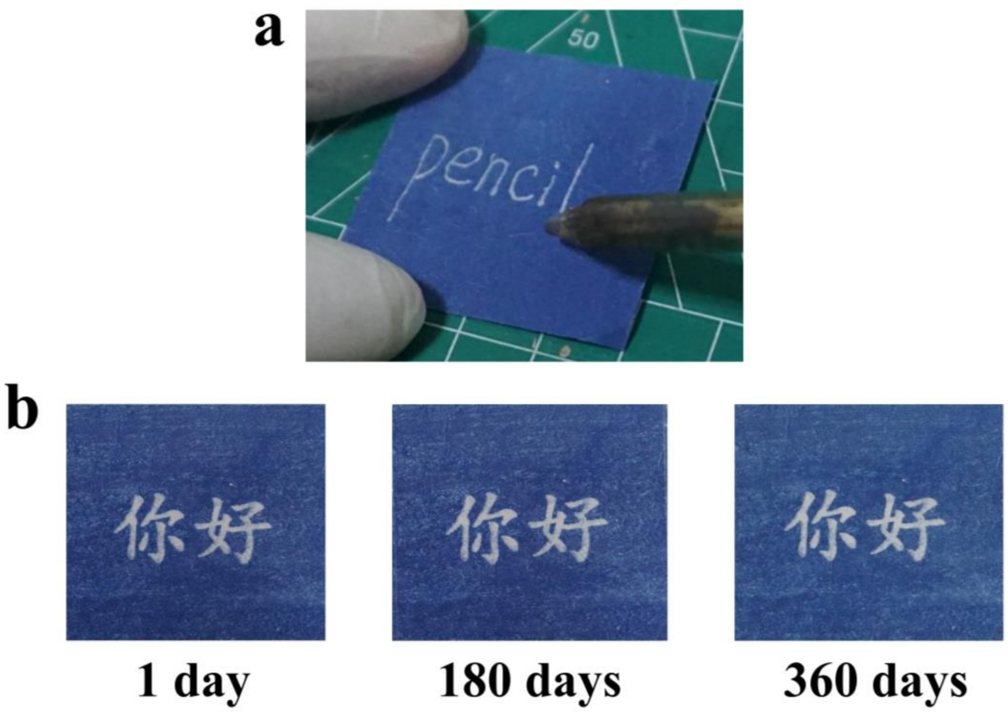

Figure S5. (a) Optical photo of handwritten English words on the rewritable TDP film by using an electrothermal pen. (b) Optical photos showing the information retention time.

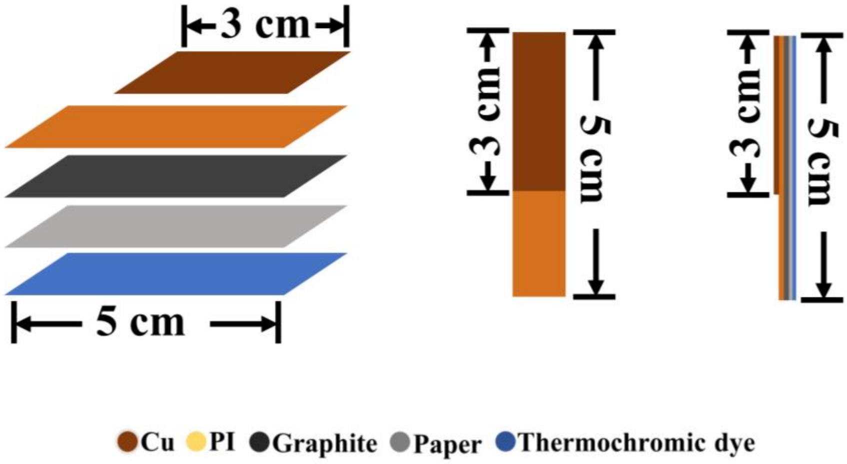

Figure S6. Structure diagram of the TDPG/PI actuator.

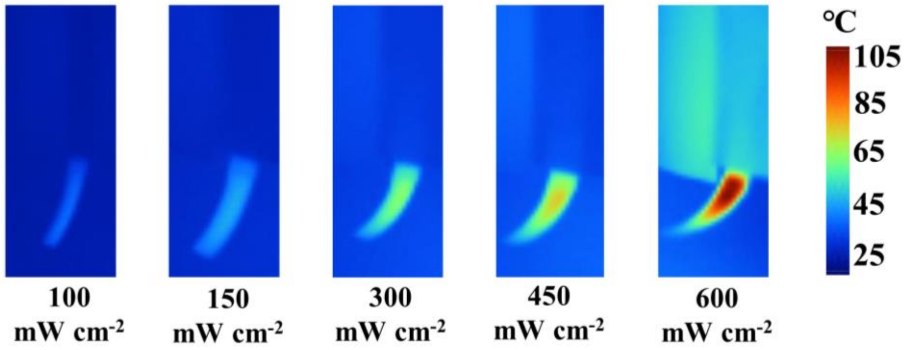

Figure S7. Infrared thermal images of the actuator under different light powers.

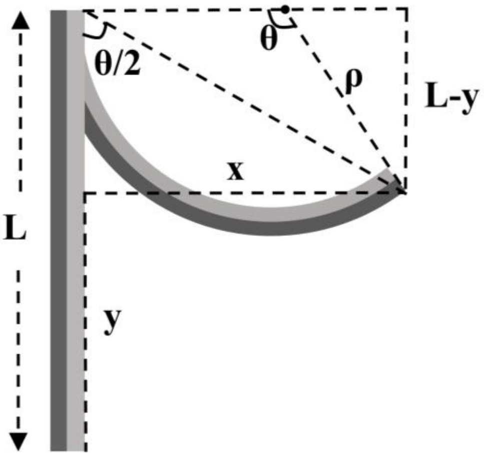

Figure S8. Light irradiated part of the actuator with correlative parameters for calculating the bending curvature.

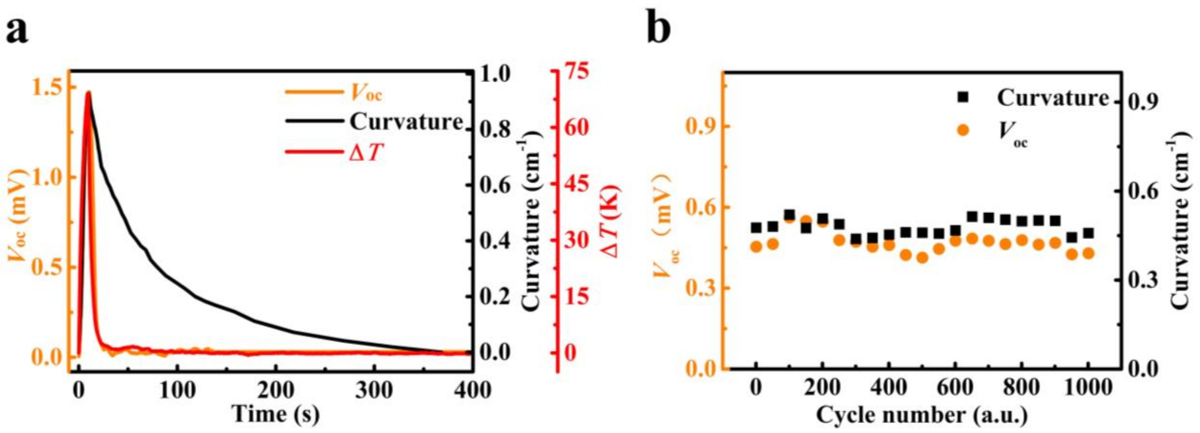

Figure S9. (a) V_{OC}, bending curvature, and ΔT of the actuator as a function of time. (b) Repeatability test of the actuator under NIR light irradiation (250 mW cm^{-2}) for 1000 cycles.

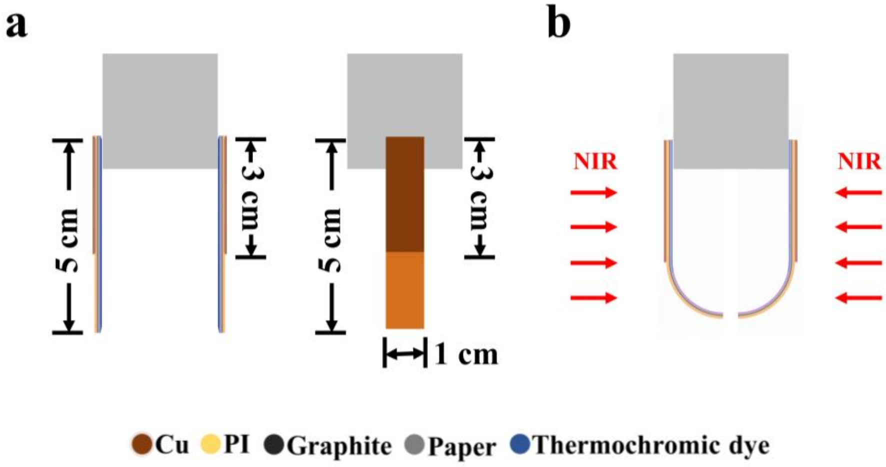

Figure S10. (a) Structure diagram of the intelligent claw. (b) Schematic diagram showing the working state of the intelligent claw.

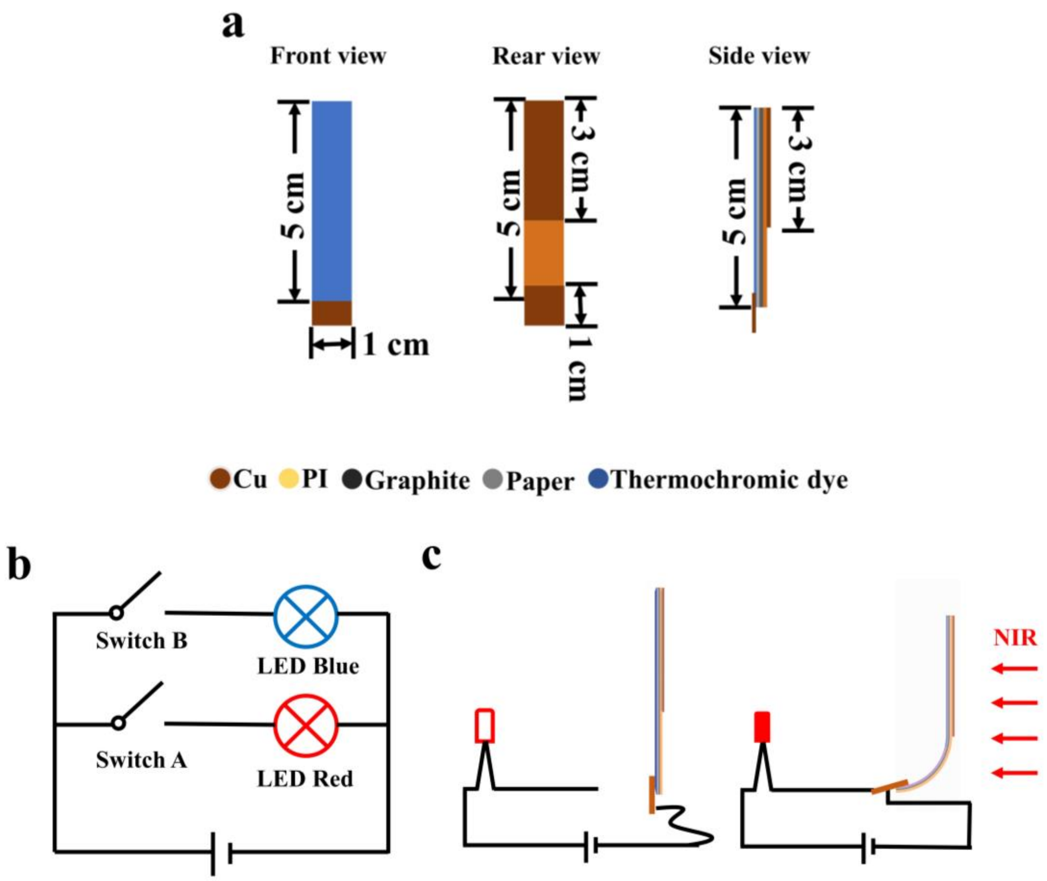

Figure S11. (a) Structure diagram of the intelligent switches. (b) Circuit diagram of the intelligent switches. (c) Schematic diagram showing the working state of the intelligent switch.

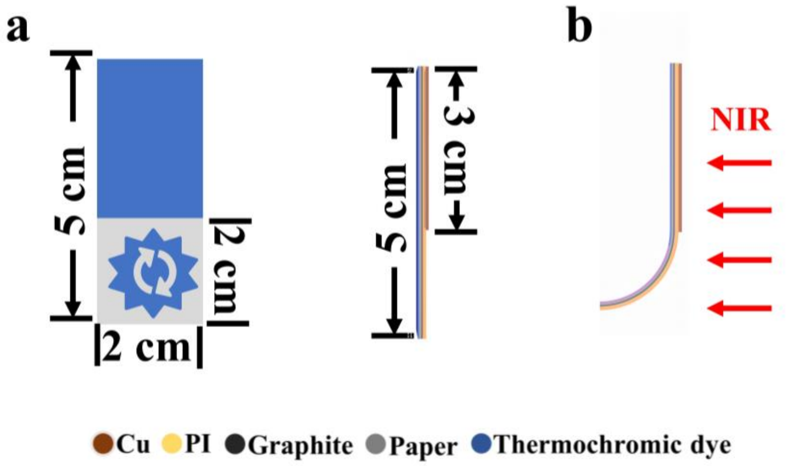

Figure S12. (a) Structure diagram of the intelligent curtain. (b) Schematic diagram showing the working state of the intelligent curtain.

Note S1.

The calculation formula of the Seebeck coefficient is deduced as

$$S = V_{OC}/\Delta T$$

where S represents the Seebeck coefficient, V_{OC} represents the open-circuit voltage and ΔT represents the temperature difference.

Note S2.

Bending curvature calculation principle of the light-irradiated part of TDPG/PI actuator. The parameters are defined as follows (shown in Figure S8):

L: The length of the light-irradiated part of actuator.

ρ: The radius of the arc of the curved light-irradiated part of actuator.

x: The horizontal displacement of the curved light-irradiated part of actuator.

y: The vertical displacement of the curved light-irradiated part of actuator.

$\theta/2$: The chord tangent angle of the curved light-irradiated part of actuator.

θ: The bending angle of the arc of the curved light-irradiated part of actuator.

The curvature is defined as the reciprocal of radius ($1/\rho$). The chord tangent angle is given by

$$\frac{\theta}{2} = tan^{-1}\frac{x}{L-y}$$

As the bending angle is given by

$$\theta = \frac{L}{y}$$

The curvature $1/\rho$ is deduced as

$$\frac{1}{\rho} = \frac{\theta}{L}$$

Therefore, the curvature of the light-irradiated part of actuator can be calculated by using the bending angle and the length of a light-irradiated part of actuator.
